# Review of studies applying Bronfenbrenner's bioecological theory in international and intercultural education research

**DOI:** 10.3389/fpsyg.2023.1233925

**Published:** 2024-01-08

**Authors:** Peiru Tong, Irene Shidong An

**Affiliations:** ^1^School of International Education, Wuhan University, Wuhan, China; ^2^Discipline of Chinese Studies, School of Languages and Cultures, The University of Sydney, Sydney, NSW, Australia

**Keywords:** bioecological model of human development, process-person-context-time (PPCT), ecological systems theory, proximal processes, international education, intercultural education

## Abstract

The Russian-born American psychologist Bronfenbrenner's bioecological perspective on human development is an ideal framework for understanding how individuals negotiate the dynamic environment and their own identities in international and intercultural education settings. However, a review of the current literature shows that most studies either adopted the earlier version of the theory (i.e., the ecological systems theory) or inadequately presented the most recent developments of the bioecological model (i.e., the process-person-context-time model). The construct of proximal processes—the primary mechanisms producing human development according to Bronfenbrenner—has seldom been explored in depth, which means the true value of bioecological theory is largely underrepresented in international and intercultural education research. This article first presents a review of studies that adopt Bronfenbrenner's theory and then offers future directions for the scope and design of international and intercultural education research.

## 1 Introduction

Bronfenbrenner's ecological theory on human development^1^ is one of the most influential and widely cited theories in the fields of human development and educational psychology (Weisner, 2008). Dissatisfied with the lack of child development research directly addressing how development is impacted by wider environments, Bronfenbrenner proposed an ecological model that can provide a framework and common language for conceptualizing the environment and identifying how the interactions and relationships among the components of the ecosystem may affect children's development (Shelton, 2019). A popular visual representation of Bronfenbrenner's ecological systems model is a diagram of the ecological system within which a toddler sits at the center, surrounded by a series of concentric circles demonstrating micro-, meso-, exo-, and macrosystems (Darling, 2007). An arrow representing the chronosystem (the influence of time) (Bronfenbrenner, 1994)^2^ is also added in some diagrams (e.g., Porter and Porter, 2020). Although Bronfenbrenner initially formulated the framework to delineate these ecological systems, he later refined it into the Process-Person-Context-Time (PPCT) model (Bronfenbrenner, 1994, 1999; Bronfenbrenner and Morris, 1998, 2006) to comprehensively consider interactions among developmental processes, contextual and individual biological characteristics, and temporal aspects.

This theory, although originated in the field of developmental psychology, is also useful for educational studies since it informs practical applications for the construction of better educational environments. In one of his earlier works, Bronfenbrenner (1977) introduced an ecological approach to education, emphasizing the dynamic relationships between learners and their environments. He challenged the traditional view of relying solely on laboratory experiments in educational research and advocated for a more holistic and ecologically valid approach to studying educational systems and processes. His focus was on the significance of real-life settings and the dynamic interactions between learners and their environments. Bronfenbrenner emphasized that understanding how individuals learn within educational settings is contingent upon the interplay between the characteristics of learners and the contexts they engage with, highlighting the intricate connections among these environments. His later article (1994), titled *Ecological Models of Human Development*, published in the *International Encyclopedia of Education*, demonstrates his considerable influence in educational research. While Bronfenbrenner's theory is most applied in child development and parental education research, it has also found use in various education-related studies, such as educational accountability (Johnson, 2008), educational transition (O'Toole et al., 2014), computer-assisted language learning (Blin, 2016), early childhood education (Tudge et al., 2017), and higher education (Mulisa, 2019). For instance, Mulisa (2019) drew inspiration from Bronfenbrenner's theory and advocated for a holistic approach that emphasizes the proximal and active interplay between students and their environments. This approach emphasizes that students' learning should not be disconnected from the social ecology of higher education. Furthermore, educational outcomes should not be attributed solely to students' competence and curriculum quality. Educators and practitioners should employ comprehensive strategies to effectively manage multilevel socioecological factors that impact students' learning.

Specifically for the field of international and intercultural education, the merits of an ecological perspective are elucidated by Elliot and Kobayashi (2019, p. 913):

[A] beautifully complex co-existence of two ecological systems develops once international students move away from their original (home country) ecological system to pursue an education in a new (host country) ecological system. Reciprocally interacting elements from various systems that affect personal, social and learning practices in particular are arguably crucial for these educational sojourners as they can lead to valuable learning opportunities as well as potential conflicts arising from competing influences emanating from the original and the new ecological systems.

Therefore, Bronfenbrenner's theory offers a nuanced and holistic framework that aids educators and policymakers in understanding, respecting, and effectively responding to the environmental complexities inherent in international and intercultural education. It helps educators appreciate the significance of diverse cultural contexts, values, and norms that influence learners, identify the crucial interactions and relationships in the intercultural settings that contribute to a student's adaptation and learning, and encourages students to engage with diverse environments for the development of intercultural competence.

This study aims to review and evaluate the application of Bronfenbrenner's developmental theory, as represented in empirical work on international and intercultural education. As noted in some critical reviews (Darling, 2007; Tudge et al., 2009, 2016; Tudge, 2016; Jaeger, 2017), the ecological theory was evolving as Bronfenbrenner continuously revised, tested and expanded his understanding of development throughout his long career (Shelton, 2019), whereas not all studies are aware of its mature version, that is, the bioecological model. Therefore, it is crucial for researchers to recognize the updated version of the theory, which reflects the most recent advance of such a powerful framework. Our objectives are threefold: First, to provide a brief overview of the evolution of the ecological theory and its historical evolution. Second, to evaluate whether the researchers in the fields of international and intercultural education adequately represented the theory in their empirical research. Third, to clarify the value of the updated version of the theory and direct future research.

We will first explain the evolution of Bronfenbrenner's ecological theory and then present some scholars' critics of its misuse in the literature. This is followed by a review and evaluation of the international/intercultural education research that has applied different versions of the theory. It will reveal that the theory is underrepresented in the current international/intercultural education literature. The paper concludes with a discussion of future directions for international and intercultural research.

## 2 The evolution and different versions of Bronfenbrenner's theory

Several scholars have provided extensive discussion on how Bronfenbrenner's ecological theory of development changed over time, from one that appears to focus primarily on contexts of development to one in which proximal processes are foregrounded (e.g., Rosa and Tudge, 2013).

In brief, Bronfenbrenner's early work in the 1970s initially spotlighted environmental contexts in human development due to the prevailing lack of attention to contextual influences within developmental psychology. Therefore, his original ecological perspective “offers a foundation for integrating context into the research model” (Bronfenbrenner, 1979, p. 21) and provides a theoretical framework that allows for the observation of a wide range of contextual influences on development (Bronfenbrenner, 1979). However, Bronfenbrenner was dissatisfied with the fact that the studies applying the model had a pervasive focus solely on contextual elements, resulting in an imbalanced focus on “context without development” (Bronfenbrenner, 1986b, p. 288). This overemphasis on context prompted a pivotal shift in the 1980s toward the integration of person, process, and time variables within the framework (Jaeger, 2017). Bronfenbrenner reformulated his model into the bioecological model by the late 1990s. This revised model positioned “proximal processes,” defined as “progressively more complex reciprocal interaction between an active, evolving biopsychological human organism and the persons, objects, and symbols in its immediate external environment” (Bronfenbrenner and Morris, 1998, p. 996), at its core. This evolution culminated in the process-person-context-time (PPCT) model, a refined iteration that accentuated the interplay of proximal processes, individual characteristics, environmental contexts, and temporal dimensions in human development (Bronfenbrenner and Morris, 1998, 2006).

While the earlier ecological model predominantly focused on environmental contexts, its emphasis on context may have led to a narrow perspective, overlooking the dynamic interplay between individuals and their immediate environment. This approach often merely compared individuals in various social or geographical contexts without delving into the developing mechanisms behind observed outcomes, assuming that all individuals in a given environment undergo the same developmental trajectory. Such an approach may, as Bronfenbrenner (1988) notes, “yield results that are not only likely to be redundant but also highly susceptible to misleading interpretations” (p. 27–28). One of the significant theoretical advancements in the bioecological model is the introduction of a critical distinction between environment and process, absent in the original ecological framework (Bronfenbrenner, 1999). While the former (environment) encompasses phenomena like mother-infant interaction and the behavior of others toward the developing person, the latter (process) is defined by its functional relationship both to the environment and to the characteristics of the developing person. The bioecological model proposes that the effects of proximal processes are more influential than those of the environmental contexts in which they occur. The evolution toward the bioecological model integrated the multifaceted interrelationships between developmental processes, individuals, contexts, and time, thereby offering a more comprehensive framework to comprehend the complexities of human development.

Bronfenbrenner (1995) highlighted Drillien (1964)'s research to exemplify the nature and scientific promise of the updated version of the bioecological model. This longitudinal study assessed factors affecting the development of children with low birth weight compared to those with normal birth weight, across different social classes over 2 years. It found that a proximal process, in this case, mother-infant interaction over time, emerges as a significant predictor of developmental outcomes, as positive maternal interaction significantly reduces behavioral issues observed in the child. The study reveals that the power of this process varies systematically based on environmental context (i.e., social class) and individual characteristics (i.e., birth weight). It highlights that the moderating effects of person and context on the proximal process of mother-infant interaction are not symmetrical. In disadvantaged environments, this interaction has the most significant effect, especially benefiting infants with normal birth weight. Conversely, in more privileged social class settings, it is low-birth-weight infants who derive the greatest advantage from maternal attention during this interaction. Therefore, one should not over- or underestimate the power of any of these factors without considering their interaction with each other. Bronfenbrenner (1999) suggests that one distinct advantage of the bioecological model, compared to other analytic designs used for analyzing environmental influences on development, lies in its recognition of the interdependency and contextual variations among influencing factors. Thus, it can address the limitations of linear multiple regression models commonly used in psychological research, which assume additive effects, and offer a more differentiated understanding of how these factors contribute to developmental outcomes by considering their synergistic effects.

The upcoming sections will outline the key elements in both the earlier and updated versions of Bronfenbrenner's theory. This will serve as a groundwork for our subsequent analysis of existing studies utilizing these distinct versions of the theory. Many studies adopting the early model of concentric circles of environments use the name ecological systems theory (EST) (e.g., Porter and Porter, 2020; Trevor-Roper, 2021; Tong et al., 2022), which is an outmoded version and a facile representation of Bronfenbrenner's theory (Tudge et al., 2009, 2016). Navarro et al. (2022) suggest that unless there are justified reasons for utilizing the earlier version, researchers should employ the latest version of the theory—the bioecological theory of human development along with the PPCT research model—and any modifications should be explicitly outlined. A summary of the key constructs in EST and the PPCT model is provided below.

### 2.1 The EST model

According to Bronfenbrenner (1986a, 1989, 1994), the ecological environment of development encompasses the four layered systems detailed in his 1979 monograph and the concept of the chronosystem introduced in his later works. Several studies (e.g., Porter and Porter, 2020; Trevor-Roper, 2021; Tong et al., 2022) examining the influence of these ecological systems on development have referred to Bronfenbrenner (1989)'s theory as EST. Although in a subsequent chapter titled “Ecological Systems Theory”, Bronfenbrenner re-evaluated his ideas from the 1979 monograph, shifting focus from context to person and process, studies using a model named after EST predominantly rely on his earlier conceptualization of ecological systems as developmental contexts. To accurately represent Bronfenbrenner's theory in the articles reviewed in this study, we use EST to denote his earlier attempt to define distinct ecological systems, namely the earlier version of his ecological theory. However, we will cite his definitions from the 1994 entry, as this is where the chronosystem was introduced as the fifth system, providing a comprehensive understanding of all five contextual influences on development as envisioned by Bronfenbrenner.

In the EST model, the development of an individual is influenced by four environmental forces, represented by nested circles (micro-, meso-, exo-, and macrosystem) and the flow of time (chronosystem). The innermost circle is the *Microsystem*, which is “a pattern of activities, social roles, and interpersonal relations experienced by the developing person in a given face-to-face setting with particular physical, social, and symbolic features that invite, permit, or inhibit engagement in sustained, progressively more complex interaction with, and activity in, the immediate environment” (Bronfenbrenner, 1994, p. 39). Settings such as family, school, peer group and workplace are all regarded as microsystems. The next layer of the circle is the *Mesosystem*, which “comprises the linkages and processes taking place between two or more settings containing the developing person” (Bronfenbrenner, 1994, p. 40), representing a system of microsystems. For instance, the linkage between school and family may affect a child's development. Then, there is the *Exosystem*, consisting of the “linkages and processes taking place between two or more settings, at least one of which does not contain the developing person, but in which events occur that indirectly influence” (Bronfenbrenner, 1994, p. 40) the person's development. One example is the relationship between a child's home and their parents' workplace. The outermost circle is the *Macrosystem*, or “the overarching pattern of micro-, meso-, and exosystems characteristic of a given culture or subculture” (Bronfenbrenner, 1994, p. 40). Finally, the *Chronosystem* “encompasses change or consistency over time not only in the characteristics of the person but also of the environments in which the person lives” (Bronfenbrenner, 1994, p. 40).

As Bronfenbrenner's thinking progressed, he called into question the overemphasis on the central role of the environment in human development and gradually made the “marked shift” to a focus on processes and a more prominent role of the developing person, reconceptualizing his theory as a bioecological model (Bronfenbrenner and Ceci, 1994). He later labeled his work a PPCT (Process-Person-Context-Time) model of development (Bronfenbrenner and Morris, 1998, p. 996). Each element of this newly evolving framework is outlined below.

### 2.2 The PPCT model

The PPCT model comprises the four defining properties of the bioecological model, emphasizing a simultaneous investigation of all these elements (Bronfenbrenner and Morris, 2006).

*Process* in the model, specifically encompassing *proximal processes*, refers to the “progressively more complex reciprocal interaction between an active, evolving biopsychological human organism and the persons, objects, and symbols in its immediate external environment” (Bronfenbrenner and Morris, 1998, p. 996) over time. Notably, the sense in which Bronfenbrenner used the term “process” (e.g., Bronfenbrenner, 1986a,b) in his earlier writings was different from the later concept of proximal process (Merçon-Vargas et al., 2020). The later formulations of proximal process illustrate the uniqueness of the concept and its importance to the theory. What is emphasized here is the joint function, involving complex interactions rather than simply the additive effects, of both human traits and the environment. It comprises the “primary mechanisms producing human development” (Bronfenbrenner and Morris, 2006, p. 795). It is crucial to clarify the distinctiveness of this concept to grasp its meaning fully and prevent confusion with related concepts such as interaction. In the context of international and intercultural education, proximal processes may involve student-teacher interactions, peer relationships, and engagement with culturally relevant learning materials. However, to qualify as proximal processes, these interactions must adhere to the criteria outlined in Bronfenbrenner and Morris (2006, p. 798). In simple terms, their measurement should encompass: (a) increasing complexity leading to either competence or dysfunction, (b) duration and frequency, and (c) reciprocal interaction (Navarro et al., 2022, p. 236).

The *Person* in PPCT model is in contrast to most developmental studies' treatment of the cognitive and socioemotional characteristics of the person as measures of developmental outcomes. It is featured both as an initial factor influencing proximal processes and as a result shaped by the interplay between person, context, and proximal processes across time. It attempts to identify process-relevant person characteristics, which was labeled person forces/disposition (differences of temperament, motivation, persistence, etc.), resources (relate to mental and emotional resources such as past experiences, skills and intelligence and to social and material resources) and demands (personal stimulus such as age, gender, skin color and physical appearance) (Bronfenbrenner and Morris, 2006). These have the “capacity to influence the emergence and operation of proximal processes” (Bronfenbrenner and Morris, 2006, p. 810). While *Context* includes the micro-, meso-, exo-, and macrosystems in the earlier EST model, the macrosystem was addressed more implicitly in writings about bioecological theory and the PPCT model (Navarro et al., 2022). The emphasis is on introducing a more significant domain within the microsystem structure, highlighting the unique impact of proximal processes involving interaction with objects and symbols, rather than solely with individuals (Bronfenbrenner and Morris, 2006). Finally, *Time* extends the original chronosystem (macro-time) to include another two levels: micro-time (what is occurring during some specific activity or interaction) and meso-time (the extent to which activities and interactions occur with some consistency in the developing person's environment) (Bronfenbrenner and Morris, 2006; Tudge et al., 2009).

All these elements in the PPCT model work interdependently and synergistically. Synergy is a key concept in the PPCT model, which refers to the cooperative action of these four elements, such that the total effect is greater than the sum of their individual effects (Navarro et al., 2022). To operationalize synergy in research, Bronfenbrenner and Morris (2006) suggest studying interactions between person and context, using multigroup models to analyze differences in developmental trajectories and outcomes across time. Navarro et al. (2022) demonstrate that the PPCT model has a minimum of four comparison groups by choosing two levels of a person characteristic and two levels of a contextual influence. These groups allow for an analysis to identify significant differences in developmental paths and outcomes among different person/context combinations over time.

Bronfenbrenner's bioecological model is no doubt a complex theory (see a summary of its constructs in Table 1). Bronfenbrenner (1986a; 1988; 1999) acknowledged the complexity and ambition of such a comprehensive paradigm, recognizing that very few researchers can address all its components simultaneously in one comprehensive analysis. It is more feasible for researchers to break down these components into smaller combinations that work together cohesively (Bronfenbrenner, 1999). He also emphasizes that the purpose of presenting this ambitious design is not to set rigid criteria for all researchers but to offer promising paradigms that generate different research questions. The goal is to alert researchers to the complexities and potential interpretative ambiguities arising from the omission of crucial elements in their selected research designs. Many scholars agree that it is not necessary to include all the factors of the PPCT model in a single study (e.g., Tudge et al., 2016; Jaeger, 2017). However, Tudge et al. (2009) asserted that to employ bioecological theory to guide a study, all four elements of the model should be present, *or* it should be clearly acknowledged why one or more of the elements are not adequately assessed in a research design, so as to preserve the integrity of the theory.

**Table 1:** Four constructs and their components in Bronfenbrenner's PPCT model [based on Bronfenbrenner and Morris (1998) and Tudge et al. (2009)].

**PPCT model constructs**	**Components**	**Meaning and features**	**Examples**
Process	Proximal process	*Reciprocal, enduring*, and *progressively more complex* interaction between an active, evolving biopsychological human organism and the persons, objects and symbols in its immediate external environment	Playing with a child, peer activities, group play, reading, learning new skills, etc.
Person	Demand characteristics	Personal characteristics that act as an immediate stimulus to another person; may influence initial interactions due to the expectations formed immediately	Age, gender, physical appearance, etc.
	Resources characteristics	Characteristics relating to mental and emotional resources; not immediately visible but sometimes are induced from the demand characteristics	Skills, intelligence, knowledge, experiences, social and material resources (such as educational background and financial and social status of family)
	Force characteristics	Cognitive, social, emotional, and motivational factors associated with temperament and personality; “active behavioral dispositions that can set proximal processes in motion and sustain their operation” (Bronfenbrenner and Morris, 1998, p. 1009)	Temperament, motivation, persistence
Context	Microsystem	The environments that the developing person engage in activities and interactions	Home, school, dormitory, peer group, classroom, etc.
	Mesosystem	Interrelations among microsystems	The relationship between family and school
	Exosystem	The contexts in which the individual whose development is not actually situated but which have important indirect impacts on their development	The parents' workplaces
	Macrosystem	A context encompassing any group whose members share value or belief systems; it envelops and influences the former systems	Culture, subculture or social structures, etc.
Time	Micro-time	What is occurring during a specific activity or some interaction	Whether an activity continues for an extensive period time without frequent interruptions
	Meso-time	The extent to which the activities and interactions occur consistently in the developing person's environment	Whether an activity occur regularly over a period of time (daily, once a week, once a month, etc.)
	Macro-time	Historical time and the life period of the individual (i.e. chronosystem)	Historical events, the distinct features of a person's different life periods, generational differences, etc.

### 2.3 Critics of the misuse of Bronfenbrenner's theory

Some review articles found that the bioecological model had been misused in many studies. These studies either cited the outmoded version or inadequately explored its components while claiming to employ the PPCT model, disregarding the resulting ambiguity due to the omission of certain constructs. For instance, Tudge et al. (2009) reviewed 25 papers published between 2001 and 2009 and showed that all but four adopted the outmoded version of the theory, which resulted in conceptual confusion and inadequate testing of the theory. After 5 years, Tudge et al. (2016) conducted a reevaluation of 20 more recent publications. The study found that although 18 of them cited the mature version (after the mid-1990s) of Bronfenbrenner's theory, only two appropriately described, tested, and evaluated the four constructs of the PPCT model. In another commentary, Tudge (2016) indicates that there are explicit and implicit ways of using Bronfenbrenner's bioecological theory: the former explicitly links research variables and methods to bioecological theory, while the latter only examines person–context interactions over time without explicitly connecting these observations to the theory's constructs. This emphasizes the necessity for the appropriate application of Bronfenbrenner's updated theory, requiring explicit recognition of its constructs as influential variables for development, as detailed in Table 1.

These reviews collectively underscore the persistent issue of inadequate adoption and exploration of the updated bioecological model, especially the nuanced constructs within the PPCT framework. The gaps identified in the literature necessitate a more thorough examination and explicit utilization of the updated theory to advance a comprehensive understanding of human development within international and intercultural education settings.

Their reviews included research up to 2016, when the model was not yet often extended to fields other than developmental science. In fact, the publications included in their reviews are mostly in the realms of family studies and child development. Therefore, this paper will review the current literature on international and intercultural education and evaluate how Bronfenbrenner's theory has been adopted in this research field.

## 3 Status of employing Bronfenbrenner in international and intercultural education: a review of current studies

**Figure 1:**
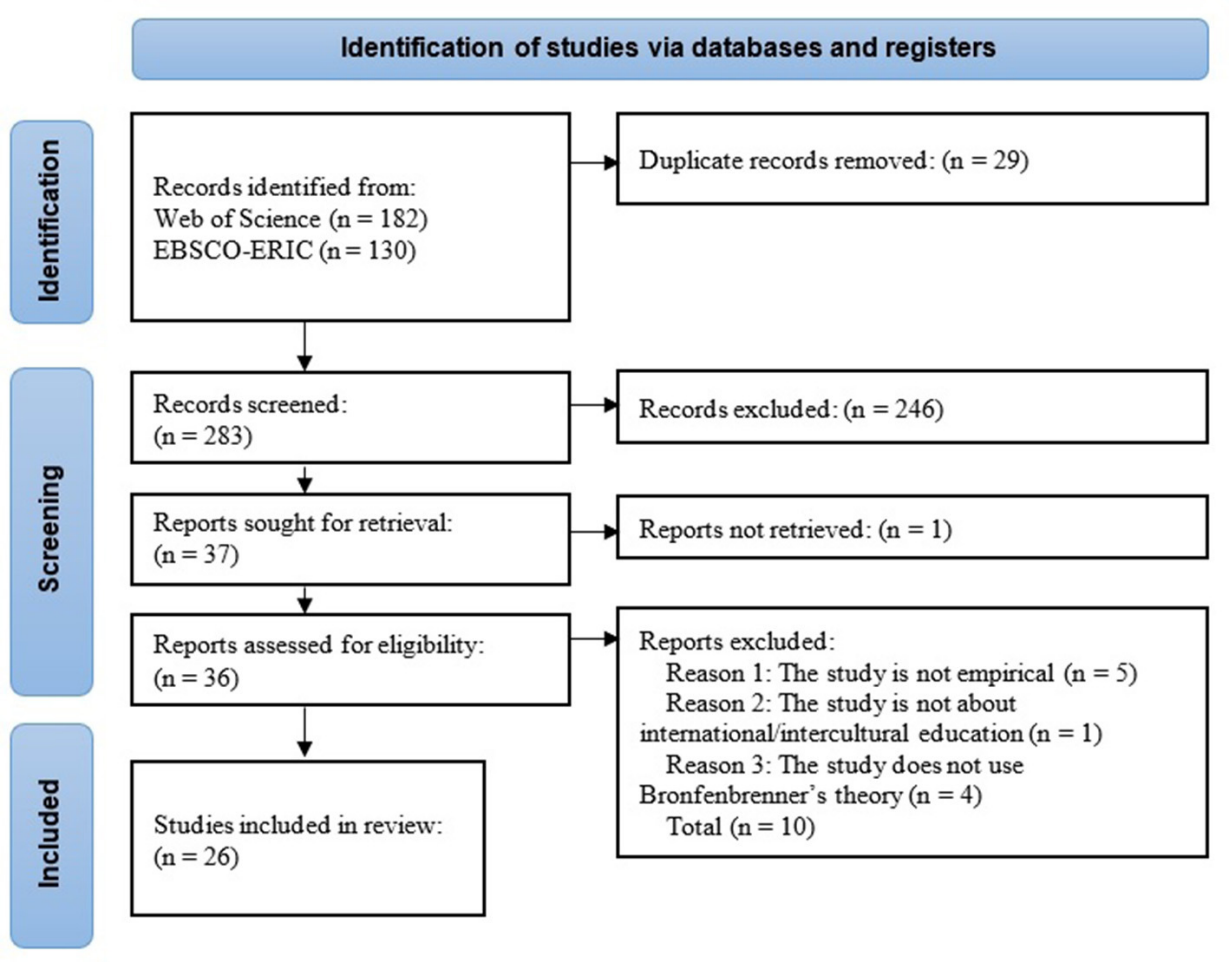
PRISMA flow diagram for searching, identifying, screening, and evaluating studies [adapted from Page et al. (2021)].

The papers to be reviewed in this section are empirical studies in the fields of international and intercultural education that claim to adopt Bronfenbrenner's ecological theory. We followed the PRISMA guidelines (Page et al., 2021) to identify and screen the papers in the databases. The PRISMA flow chart is presented in Figure 1.

The terms used for searching studies using Bronfenbrenner's theory followed Tudge et al. (2009) and Jaeger (2017): Bronfenbrenner/bioecological/ecological systems theory/process–person–context–time/PPCT. We also used the keywords international/intercultural/study abroad/exchange/mobility/overseas to constrain the research field to international or intercultural education. We searched the Web of Science (WoS) databases (SSCI/SCI-Expanded/ESCI/A&HCI) (up until 12 September 2023) to ensure that the articles obtained were of good quality. We also conducted searches using a specialized database, EBSCO-ERIC (Educational Resource Information Center), up until September 12, 2023, to identify any additional studies specifically relevant to education. The following inclusion criteria were applied to the initial searches in both databases: (a) studies published in peer-reviewed academic journals, (b) studies published in English, and (c) empirically designed studies, excluding other types such as editorials and review articles. Additionally, we limited the WoS Categories to psychology, education, and related fields like linguistics and social sciences. We also included multidisciplinary categories to retrieve potential studies. Detailed search strategies, including filters and limits used for both databases, are specified in the Appendix. These searches yielded 182 results in the WoS databases and 130 in the ERIC database, totaling 283 after discarding duplicates.

The two researchers screened these records, encompassing titles, abstracts, and keywords, to determine their eligibility for further evaluation. Initially, they conducted independent screenings, resolving disagreements through collaboration. Subsequently, studies were manually eliminated if they: (a) were non-empirical, (b) did not pertain to intercultural or international education (for example, studies merely containing the keyword “international” but not related to international education), and (c) did not apply Bronfenbrenner's theory (for instance, studies related to ecological and environmental education containing the keyword “ecological” but not employing an ecological perspective to investigate educational issues). Studies with uncertainties regarding their article type, research scope, or theoretical perspective were reserved for further examination. Following this screening, 37 reports were initially considered for retrieval, although the authoritative versions of one article could not be retrieved. The researchers then thoroughly examined the full papers of the remaining 36 studies, discarding ten articles based on the aforementioned criteria. Consequently, 26 studies remained for inclusion in this review, as summarized in Table 2.

**Table 2:** Studies on international and intercultural education employing Bronfenbrenner's ecological theory reviewed in this study.

**References**	**Phenomena under study**	Bron. cite^*^	**Naming of the theory**	**Research method**
Bhowmik et al. (2018)	Acculturative stress and coping strategies among mainland Chinese university students in Hong Kong	2006	Socioecological model	Focused group interviews
Chkaif et al. (2022)	African students' mobility to China	1979	Ecological systems theory	Survey and interview
Conceição et al. (2021)	Brazilian Students Studying in the United States	1994	Ecological systems theory	Open-ended, self-reflective online questionnaire
Elliot and Kobayashi (2019)	Supervisors' interactions with international students	2005	Bio-ecological theory of human development	Interview
Elliot et al. (2016a)	International PhD students' academic acculturation	2005	Bio-ecological theory of human development, Bio-ecological systems theory	Visual metaphor approach
Elliot et al. (2016b)	International students' academic acculturation	2005	Bio-ecological theory of human development, Bio-ecological systems theory of human development, Ecological systems theory	Interpretative phenomenological approach
Emery et al. (2020)	Parent perspectives on schooling experiences of internationally adopted youth with disabilities	2006	Bioecological systems model	Qualitative analysis; questionnaire consisting of open-ended items
Jessup-Anger and Aragones (2013)	Students' peer interactions during a short-term study abroad	1993	Ecological systems theory	Constructivist approach; qualitative case study design; observation, interviews, and document review
Li and Que (2016)	Integration and career challenges of newcomer youth in Canada	1979	Ecological systems theory	Qualitative case study design; one-on-one interviews
Liu et al. (2022)	Academic career development of Chinese returnees with overseas PhD Degrees	2006	Bioecological model of human development	Semi structured interviews; a narrative approach
Marangell (2023)	Students' experiences of an internationalized university	2005	Ecological model of human development	Case study design; mixed-methods approach; questionnaire; interviews
McBrien (2011)	Refugee mothers' involvement in their children's schools	1979	Ecological systems model/theory	Focus group interviews
Merchant et al. (2020)	School administrators' responses to refugee students in their rural communities	1979	Ecological systems theory	Individual and focus group interviews; document analysis
Ngo et al. (2022a)	Professional development experiences of Vietnamese tertiary English as a foreign language lecturers	1979	Ecological systems theory	Semi-structured interviews; document analysis; a phenomenological approach
Ngo et al. (2022b)	Contextual influences on the professional development experiences of Vietnamese tertiary English as a foreign language lecturers	1979	Ecological systems theory	Semi-structured interviews; document analysis; a phenomenological approach
Porter and Porter (2020)	Japanese students' decisions to study abroad	1986	Ecological systems theory	In-depth interviews
Rokita-Jaśkow et al. (2023)	School socialization of bi/multilinguals in the eyes of English as a foreign language teachers	1979	Ecological perspective	Semi-structured interviews analyzed using a content analysis method
Suárez-Orozco et al. (2010)	Variations in academic trajectories amongst immigrant youth	1977	Ecological systems framework	Longitudinal study; interviews involving different question formats (open-ended, fill-in-the-blank; Likert scales, etc.); laten class growth curve analysis and multinomial logistic regressions
Taylor and Ali (2017)	Factors that influence meaningful learning and assimilation	1993^**^	Ecological theory of human development	Timeline interviews analyzed thematically
Tong et al. (2022)	An Australian-Chinese student's study abroad experience in Hong Kong	1994	Ecological systems theory	Interviews and reflective journals; narrative analysis
Trevor-Roper (2021)	International academic affiliations	1979	Ecological systems theory	Interview-based study
Vardanyan et al. (2018)	A Syrian immigrant child's language acquisition and culture adaptation in the United States	1994	Bio-ecological theory of child development; ecological theory/model	Ethnography and case study approaches; semi-structured interviews; observations; field notes
Winer et al. (2021)	Acculturation experience of children of international migrants	1979	Ecological systems theory	Phenomenological approach; implication analysis of children's artwork; observation
Xu and Tran (2022)	Chinese international doctoral students' navigation of a disrupted study trajectory during Covid-19	2006	Bioecological systems theory	One-on-one semi-structured interview
Xu et al. (2021)	Negative and positive forces that influence students' developmental trajectories during their doctoral education	2006	Bioecological systems theory	Volunteer-employed photography
Zhang (2018)	Academic advising with international students	1992	Ecological model	A phenomenological research design; interview

^*^Most recent Bronfenbrenner work cited by the author(s). ^**^Although there is a citation of Bronfenbrenner's work in 2009, the original publication dates back to 1979, so the most recent work cited is Bronfenbrenner's publication in 1993.

Some initial observations can be made from Table 2. First, although we did not set the starting year for our search period, most eligible studies were published in the recent decade, suggesting that Bronfenbrenner's theory has been applied only to the field of international and intercultural education quite recently. Second, 18 studies cited Bronfenbrenner's work before the mid-1990s or named the theory EST or ecological model/theory; thus, they did not use the mature version. Another seven studies cited his work after 2000 and used the term “bioecological” (Bronfenbrenner and Morris, 1998; Bronfenbrenner, 2005), demonstrating the researchers' awareness of the recent update on the framework. The remaining study (Bhowmik et al., 2018), although cited Bronfenbrenner and Morris's (2006) work, did not use the term “bioecological” (instead, they named the theory “a socioecological model”)^3^. Third, most studies relied solely on qualitative methods to collect and analyze data.

The studies can be grouped into several categories according to how Bronfenbrenner's theory is used: loosely connected to Bronfenbrenner's theory, EST-based, and based on the updated version of the bioecological model (see detailed categorization in Table 3). Recognizing that the application of Bronfenbrenner's theory is still in its infancy in international and intercultural education research, our objective is not to critique individual articles but to understand the extent to which the empirical studies we reviewed reflect the recent development of the theory.

**Table 3:** Categorization of the studies reviewed.

Loosely connected		**Suárez-Orozco et al., 2010; Bhowmik et al., 2018; Elliot and Kobayashi, 2019; Trevor-Roper, 2021**
EST-based	Partial adoption	Jessup-Anger and Aragones, 2013; Elliot et al., 2016b; Li and Que, 2016; Taylor and Ali, 2017; Vardanyan et al., 2018; Emery et al., 2020; Merchant et al., 2020; Porter and Porter, 2020; Winer et al., 2021; Rokita-Jaśkow et al., 2023
	Full adoption	McBrien, 2011; Zhang, 2018; Ngo et al., 2022b; Tong et al., 2022
	Extended adoption	Elliot et al., 2016a; Conceição et al., 2021; Chkaif et al., 2022; Ngo et al., 2022a; Xu and Tran, 2022; Marangell, 2023
Based on the updated version		Xu et al., 2021; Liu et al., 2022

### 3.1 Studies loosely connected to Bronfenbrenner's theory

Four studies (Suárez-Orozco et al., 2010; Bhowmik et al., 2018; Elliot and Kobayashi, 2019; Trevor-Roper, 2021) are only loosely connected to Bronfenbrenner's theory, although they cite his work, either the early or the mature version. These studies only mention Bronfenbrenner in their papers but have not systematically applied his theory. Bhowmik et al. (2018) cite Bronfenbrenner's work without using the constructs of his theory for data analysis. Elliot and Kobayashi (2019) only mention the coexistence of two ecosystems of international students but have not specified the components in each layer of the ecosystem. Suárez-Orozco et al. (2010) reference Bronfenbrenner's early work (Bronfenbrenner, 1977) to highlight the significance of contexts and characteristics affecting students' performance. However, while their research explores the influences of school, family, and individual characteristics on immigrant children's academic trajectories, it lacks a systematic foundation based on Bronfenbrenner's theory. Moreover, the study findings are not explicitly interpreted in connection with Bronfenbrenner's framework. Similarly, Trevor-Roper (2021) only briefly discusses that the EST model is helpful in appreciating the complexity of higher education environments in international education but does not follow the model's constructs to frame the data analysis.

In other words, Bronfenbrenner's theory only serves as an overarching philosophical perspective rather than an operational model that guides detailed data analysis procedures in these studies. Such an approach partially overlaps with Tudge (2016) description of the “implicit way” of using Bronfenbrenner's theory, which only examines person–environment interactions and the complexity of the environment. This can be problematic since it oversimplifies the richness of Bronfenbrenner's theory and does not sufficiently demonstrate its value for international and intercultural education.

### 3.2 Studies based on EST

Twenty studies (McBrien, 2011; Jessup-Anger and Aragones, 2013; Elliot et al., 2016a,b; Li and Que, 2016; Taylor and Ali, 2017; Vardanyan et al., 2018; Zhang, 2018; Emery et al., 2020; Merchant et al., 2020; Porter and Porter, 2020; Conceição et al., 2021; Winer et al., 2021; Chkaif et al., 2022; Ngo et al., 2022a,b; Tong et al., 2022; Xu and Tran, 2022; Marangell, 2023; Rokita-Jaśkow et al., 2023) are based on the early version, that is, the EST model, although some of them cite Bronfenbrenner's later work and use the term “bioecological.” Three sub-categories can be identified: partial adoption, full adoption, and extended adoption of EST.

#### 3.2.1 Partial adoption of EST

Ten of the 20 studies, including Jessup-Anger and Aragones (2013), Elliot et al. (2016b), Li and Que (2016), Taylor and Ali (2017), Vardanyan et al. (2018), Emery et al. (2020), Merchant et al. (2020), Porter and Porter (2020), Winer et al. (2021), and Rokita-Jaśkow et al. (2023) are all classified as partial adoption.

Elliot et al. (2016b)'s study on international students' academic acculturation focuses exclusively on the chronosystem in the EST model. They identify different forms of personal transition, societal transition, and academic transition of international students. Conversely, Emery et al. (2020)'s study explores the experiences of internationally adopted youths across various systems (micro-, meso-, exo-, and macrosystems), with a specific focus on the mesosystem, where schools are pivotal in providing support. Their study does not address the chronosystem.

Jessup-Anger and Aragones (2013) primarily delve into the influence of developmentally instigative characteristics (Bronfenbrenner, 1993) on interactions of study abroad students in host countries, discussing micro- and mesosystems. In Merchant et al. (2020)'s work on refugee students, they highlight the mesosystem (interactions between families, peers, and schools) and exosystem (neighborhood and community organizations) as influential in shaping students' wellbeing. Li and Que (2016)'s study focuses on integration challenges faced by newcomer youths in a Canadian city, emphasizing themes related to the exosystem (public transportation), microsystem (family support, social interaction), and individual factors like language barriers and job pressures. Porter and Porter (2020) analyze factors influencing Japanese college students' decisions to study abroad, considering various ecosystem layers (micro- and mesosystems as immediate environments, and exo-, macro-, and chronosystems as distant environments). They omit the mesosystem due to limited participant input. Conversely, Taylor and Ali (2017) incorporate the mesosystem while excluding the exosystem in their examination of international students' adjustment to studying in the UK. They do not distinctly explain the rationale for excluding the exosystem, potentially due to data limitations.

Rokita-Jaśkow et al. (2023)'s study on the school socialization of bi/multilingual children examines the microsystem (teachers), mesosystem (classmates and parents), and exosystem (representatives of the education system). However, it omits the macro- and chronosystems within the EST framework without providing an explanation. Vardanyan et al. (2018) employ Bronfenbrenner's EST concepts in their data analysis, emphasizing the micro- and mesosystems, with limited focus on the chronosystem. In contrast, Winer et al. (2021) explore immigrants' children's sense of belonging within the microsystem (their rooms in their homes), mesosystem (a shared living building), and macrosystem (their neighborhood). However, they do not introduce or investigate the exo- and chronosystems.

These studies collectively illustrate that the EST is a multifaceted model, demanding multiple investigations to comprehensively explore the entire ecological system (Elliot et al., 2016b). However, there is a need for more explicit justification when certain constructs within the model are excluded from analysis, as this exclusion affects the overall comprehensiveness of the theory.

#### 3.2.2 Full adoption of EST

Four studies investigate all the components of EST. McBrien (2011) delves into the challenges encountered by refugee mothers as they adapt to settled lives and explores their children's schooling experiences in the context of all the components within the EST. Ngo et al. (2022b) investigate the impact of contextual factors on the professional development experiences of Vietnamese English as a foreign language lecturers across different contextual levels within the EST model. Tong et al. (2022) use the EST model to offer a visual metaphorical illustration of the major themes at each level of an Australian–Chinese student's developmental ecosystem in Hong Kong and tease out the risk and protective elements in this ecosystem that influenced the student's developmental trajectory. Zhang (2018) examines how academic advising with international students was shaped by individual backgrounds and multiple layers of environmental influences.

These studies meticulously examine each construct of EST within the context of international and intercultural education and demonstrate the relevance of the model in fostering positive interactions in intercultural settings.

#### 3.2.3 Extended adoption of EST

Six articles extend the model to some degree. Chkaif et al. (2022) combine EST with Yu et al. (2021) to generate a refined model for international education, with the macrosystem being revised to include the global dimension. Conceição et al. (2021) expand upon the investigation of the chronosystem within the EST by integrating transformative learning theory to illustrate the personal growth and development of study abroad students over time. Elliot et al. (2016a) propose an academic acculturation model illustrating the transition between two ecosystems of a study-abroad sojourn. Marangell (2023)'s study on students' experiences at an internationalized university applies a person-in-context (PiC) model (Volet, 2001), which adapts Bronfenbrenner's EST. The PiC model centers on the “experiential interface,' where individual and environmental dimensions interact, and explores how congruence between these dimensions fosters motivated and productive learning. Ngo et al. (2022a) incorporate EST into an integrated framework for effective professional development, encompassing three dimensions: context, content, and process. Finally, Xu and Tran (2022) extend the investigation of the person at the center of EST by employing the needs–response agency theory.

These studies provide nuanced perspectives that enhance EST's applicability in international and intercultural education and underscore the importance of the continuous evolution of the theory to address the complexities of educational systems in an increasingly interconnected world. However, the expansion of the theory also introduces extra complexity and challenges in operationalizing and measuring the constructs, and care should be taken to disentangle various factors.

The EST-based studies reviewed above offer valuable insights into international and intercultural education within Bronfenbrenner's early EST model by discussing various aspects, such as the impact of cultural contexts, policy frameworks, academic transitions, peers, and advisors, all of which are crucial in understanding educational experiences in diverse cultural settings. Nevertheless, the absence of the PPCT model in these studies limits the exploration of the dynamic processes and interactions between individuals, their contexts, and the outcomes of international and intercultural education.

### 3.3 Studies based on the updated version of the bioecological model

The final two studies (Xu et al., 2021; Liu et al., 2022) was more pertinent to the bioecological model, although they do not mention the PPCT model. They differ from other studies reviewed above in that they not only recognize the existence of the mature version of Bronfenbrenner's theory but also employ it to guide their data analysis. For instance, Liu et al. (2022) state that while they acknowledge the influence of ecological systems in Bronfenbrenner's early model, they further embrace his later theoretical development of the bioecological system, which considers the individual as an active agent in proximal processes. Xu et al. (2021) also comment in their article that previous studies applying Bronfenbrenner's theory to address academic acculturation neglect a thorough identification of individual and contextual forces and fail to delineate the dynamic interactions between them. Therefore, both studies employ the updated version of the theory by highlighting how the “person” constructs (dispositions, demands, and resources) interact with environmental contexts to shape development. Liu et al. (2022) investigate the academic career development of Chinese returnees with overseas PhDs. (CROPs) and find that preferences for stability (dispositions), social networking establishment and maintenance (demands), and a lack of experience with local academic and publication cultures (resources) are important factors. Xu et al. (2021) examine Chinese doctoral students' international education experiences in Australia and suggest that personal characteristics, such as inward management practices (dispositions), social networking maintenance (demands), research outputs, and health status (resources), are the engine of development.

These two studies contribute to the field of international and intercultural education by recognizing and utilizing this updated version of Bronfenbrenner's theory. However, both studies only briefly mention the concept of “proximal processes”—the core of the mature version of the bioecological model—without identifying what they were and how they contributed to development. For instance, Xu et al. (2021) acknowledge that the core driving force in the bioecological system relies on PhD students' ability to initiate their autonomy as they negotiate, utilize, and create resources for their development in both their home and host environments. However, they state that “a fine-grained elaboration of these practices is neither the focus of this study nor possible to accomplish in a piece of this length” (p. 1354). Notably, these practices embody potential proximal processes of interest in the bioecological model. Furthermore, neither study adopts a PPCT design. Despite acknowledging the interplay between Person and Context factors in development, the absence of specifying the proximal processes mediating these effects limits the studies from achieving the synergistic design envisioned in Bronfenbrenner's bioecological theory.

### 3.4 Summary

This review indicates that the application of Bronfenbrenner's bioecological theory in international and intercultural education research is still in its infancy. Most studies have adopted the elements of EST to explain international and intercultural contexts for education, while the updated version of PPCT has been inadequately explored. In the study where PPCT is referenced (Emery et al., 2020), it is mentioned as background information rather than being utilized as a framework for interpreting empirical findings. Proximal processes, crucial in the PPCT model and critical to international and intercultural education, have seldom been explored in depth, which means that the true value of Bronfenbrenner's theory is largely underrepresented.

## 4 Conclusions and future directions

Bronfenbrenner's theory has undergone continuous refinements and reformulations over time and has evolved from an ecological to a bioecological theory, incorporating a four-element model (PPCT), in which the proximal process is given pride of place (Tudge et al., 2016). Previous reviews have criticized the misuse or partial representation of Bronfenbrenner's theory in the field of developmental science, especially in family studies and child development (Tudge et al., 2009, 2016; Tudge, 2016). However, as Bronfenbrenner's theory has become influential in other fields in recent years, how the theory has been employed in these fields is a compelling question. This review addresses this issue and provides new insights for scholars in the field of international and intercultural education who are interested in applying Bronfenbrenner's bioecological theory.

In international and intercultural education, students experience a collide of at least two ecosystems consisting of complex elements and relationships. Therefore, Bronfenbrenner's bioecological perspective on human development is an ideal framework for understanding how an individual negotiates the dynamic environment and their own identity in these intercultural settings (Elliot and Kobayashi, 2019; Xu et al., 2021; Xu and Tran, 2022). The theoretical merit of the PPCT model is that it allows researchers to capture the dynamics and relationships between organisms and environments rather than presenting the developing person and influencing contextual factors discretely. Although the fields of education and development have benefited from a focus on contextual influences on human development situated within the early ecological model of Bronfenbrenner (1979), the PPCT model can inspire new ways of thinking about contextual influences (Bronfenbrenner, 1999). Firstly, this model refines the concept of microsystem by emphasizing proximal processes within these microsystems, identifying them as pivotal mechanisms through which development occurs. Secondly, the PPCT model posits that these proximal processes act as moderators, shaping the impact of contextual influences. Bronfenbrenner's work underlines that while contexts exert significant influence, the quality and nature of proximal processes within these contexts can moderate or amplify their effects on individual development. This model thereby deepens our understanding by emphasizing the interactive and dynamic nature of contextual influences.

However, a review of the existing literature indicates that when Bronfenbrenner's theory is applied to international and intercultural education research, either the earlier version of EST is used or the mature version is only partially applied, without paying the due attention to proximal processes and how they are jointly influenced by the personal characteristics, various levels of contexts and time variables. Based on this review, we propose the following future directions for international and intercultural education research regarding theoretical perspectives and methodological designs.

### 4.1 Future directions for theoretical perspectives

For scholars seeking to apply Bronfenbrenner's theory in their empirical studies, we propose two recommendations.

First, consistent with other scholars' previous reminders (e.g., Tudge et al., 2009; Rosa and Tudge, 2013; Navarro et al., 2022), we also emphasize on the importance for studies to clearly specify which version of the theory they are adopting and to provide a rationale for their choices. This clarity will help avoid the “two-fold disservice” pointed out by Tudge et al. (2009, p. 198) and thus allow for an accurate understanding of the theoretical framework, enhance the comparability and consistency of research findings, contribute to the cumulative knowledge in the field, and facilitate the comparison and synthesis of findings across studies. Scholars may choose to adopt the early version of the theory, the EST model, if their study primarily focuses on environmental factors, or they may opt for the mature version of the theory, the PPCT model, if they aim to highlight the crucial impact of proximal processes, and their dynamic interplay with individuals, their characteristics, and their immediate and remote environmental contexts and historical time. However, it is misleading if a study claims to use Bronfenbrenner's “bioecological” theory and only refers to the EST model without acknowledging the updated PPCT model or if a study only partially adopts some constructs in either model without explaining the rationale. Researchers should carefully consider whether a theory is appropriately represented to ensure the credibility and robustness of the research.

Second, to advance the field of international and intercultural education research, we suggest employing the mature version of Bronfenbrenner's bioecological theory, which emphasizes the significance of proximal processes. This shift in emphasis can contribute to a more comprehensive understanding of how individuals actively engage with their educational environments. In child development research, a study involving the PPCT model may examine how regularly occurring parent–child interactions, such as joint storybook reading (e.g., Barnyak, 2011), are influenced by important characteristics of the child and some relevant aspects of the context (Tudge et al., 2009). Similarly, in international and intercultural education, researchers can gain deeper insights into the everyday activities that shape individuals' development in diverse cultural contexts by focusing on proximal processes. For example, interactions with local people and peers are two different types of proximal processes that an international student may encounter in the host country, which may have either positive or reverse effects on their development. Merçon-Vargas et al. (2020) further propose the notion of inverse proximal processes to expand the conceptual framework of proximal processes and to address the potential negative impact of certain interactions and activities on human development, particularly in disadvantaged environments. This concept suggests that in disadvantaged environments, detrimental or dysfunctional interactions occurring regularly over extended periods of time are linked to higher levels of dysfunction and lower competency. The notion of inverse proximal processes allows for a more inclusive and expansive use of Bronfenbrenner's bioecological theory.

One question concerns the identification and measurement of proximal processes for investigation, and there is no straightforward answer as Bronfenbrenner did not provide a definitive guideline. In Drillien (1964) study, Bronfenbrenner identified mother-infant interaction as a proximal process, measured by maternal responsiveness through family observations and interviews. He highlighted that a more comprehensive understanding of proximal processes should also encompass the infant's responsiveness to changes in the mother's behavior, reflecting the reciprocal nature of proximal processes. For Small and Luster (1990)'s study, parental monitoring was identified as a proximal process, assessed through a questionnaire on adolescents' perceptions of parental efforts to stay informed and set limits on their activities outside the home. These examples indicate that diverse tools such as observations, interviews, and questionnaires can measure proximal processes, provided they align with the concept's definition. Therefore, we concur with Navarro et al.'s (2022) guideline that measures of proximal processes should consider: (a) increasing complexity over time (either inverse or positive); (b) reciprocity between the developing individual and the interacting person(s)/object(s); and (c) duration (i.e., microtime) and frequency (i.e., mesotime) to ensure regular occurrence over an extended period. We also regard it appropriate to design measurement methods tailored to specific research questions.

Previous literature in international and intercultural education has identified key processes contributing to student development, such as interacting with native speakers (Campbell, 2011), engaging in cultural activities (Isabelli-García, 2006), and attending international courses (Jiang et al., 2023). Therefore, studies aiming to investigate these as proximal processes need to examine the level of complexity, mutual engagement, and regularity of these activities and their changes over time. For instance, a study on interacting with native speakers might scrutinize conversation topics for complexity, native speakers' responses for reciprocity, and the duration and frequency of these conversations for regularity. Meanwhile, research on cultural activities might explore the complexity and regularity of different tasks within these activities and how they stimulate subjects' “attention, exploration, manipulation, elaboration, and imagination” (Bronfenbrenner and Morris, 2006, p. 798).

By following these recommendations, scholars can enhance the applicability and relevance of Bronfenbrenner's theory in the context of international and intercultural education and contribute to the advancement of the empirical field.

### 4.2 Future directions for methodological designs

Future research in international and intercultural education can benefit from several key methodological considerations. First, studies framed by the bioecological perspective should aim to meet the requirements of a PPCT study design. Bronfenbrenner did not conduct his own research using the PPCT model; instead, he referenced other scholars' work to showcase his concepts. Therefore, interpreting and applying the PPCT model can pose challenges. Navarro et al. (2022) provide a detailed guideline of how a study should address all the PPCT components to ensure that its design enables a “Bronfenbrenerian synergistic analysis” (p. 240). For instance, the guidelines highlight that PPCT studies must be longitudinal, as the outcome must be measured at a developmentally relevant time point after the proximal process(es), which, as another requirement, should be examined regarding increasing complexity, reciprocity, and duration and frequency. They also note that when applying the bioecological theory and the PPCT model, it is crucial to carefully choose the pertinent elements of person, context, process, and time by thoroughly reviewing empirical studies and pertinent theoretical perspectives. Meanwhile, the synergy among these components should be elaborated, which means the “cooperative action of discrete agencies such that the total effect is greater than the sum of two or more effects—taken independently” (Bronfenbrenner and Morris, 2006, p. 800), suggesting the use of multigroup models (Navarro et al., 2022). Navarro et al. (2022) demonstrate that the PPCT model allows for the comparison of at least four groups based on different person/context combinations. Quantitative research can use mediational models to assess developmental differences over time, while qualitative researchers will also need to ensure their individual participant selection meets these criteria.

By aligning the research design with the principles and guidelines outlined, researchers can ensure a comprehensive and systematic examination of the four components in the PPCT model. Let us consider how the studies based on the updated version of the bioecological model reviewed above can be redesigned to more closely approximate the PPCT design. For instance, consider Liu et al. (2022)'s research where they identify several factors influencing CROPs' career development, including: (a) the lack of recent experience and familiarity with local academic and publication cultures hindered career development, (b) interactions in the microsystem with senior leaders, line managers, and colleagues had negative impacts, leading to academic pressure and mental health concerns, and (c) the macrosystem of Chinese higher education, driven by the ambition to establish world-class universities, shaped the microsystem's interactional hostility due to marketization and globalization influences in international higher education. Building on these findings, a research approach based on the PPCT model might explore the relationships between these factors using a longitudinal design. Subjects could be categorized along two dimensions: CROPs' familiarity with local academic publication cultures (a Person factor) and the type of university they are working in (a Context factor). This differentiation might involve a national funded university aiming for higher rankings in the Chinese higher education system, reflecting a specific macrosystem, and Sino-Foreign Joint Venture Universities, which mirror a distinct macrosystem akin to Western educational culture. Interactions with senior leaders might serve as a proximal process, varying in positivity or negativity. Developmental outcomes could encompass academic competence (e.g., publications, grants, self-efficacy) and dysfunction (e.g., stress, mental health issues). One potential outcome of such a design might reveal that positive interactions with senior leaders foster academic competence among those familiar with local academic cultures and alleviate academic stress among those less acquainted. However, these interaction effects may differ between national funded universities and Sino-Foreign Joint Venture Universities. This assumption draws from Liu et al. (2022)'s evidence highlighting that in the Chinese culture, “Big Figures” (Da Lao in Chinese) impact resource allocation, potentially influencing the culture of national funded university more significantly. Such insights would deepen our understanding of the intricacies within intercultural settings in higher education.

The PPCT model also offers a means to address conflicting research findings in international and intercultural education research. In the sphere of study abroad research, for instance, there has been extensive exploration of outcomes and influencing factors such as living conditions (Allen et al., 2006), social networks (Magnan and Lafford, 2012), and duration of stay (Dwyer, 2004). However, this body of work often yields conflicting or overly generalized conclusions (Pinar, 2016). Take living condition as an example, while some studies emphasize the positive influence of living with host families on language and intercultural competence development (Allen et al., 2006), others, such as those by Isabelli-García (2006) and Jackson (2009), highlight potential negative effects if interaction with the family is troubled or almost non-existent. Critiques by Coleman (2013) emphasize the oversight of contextual uniqueness and individual variables in demonstrating study abroad benefits, echoed by Ushioda (2009) emphasis on the varied impact of social or individual factors. In considering the bioecological model, the study abroad setting does not predict learning outcomes in isolation; rather, it is the activities (proximal processes) in which the students engage that wield greater influence. Therefore, employing a PPCT design could effectively address these controversies by illuminating how varied proximal processes produce differed developmental outcomes as a joint effect of individual characteristics and contextual factors.

Let us envision a hypothetical study that utilizes the PPCT design to navigate the controversial outcomes regarding the influence of host family contexts on students' development of intercultural communication competence. Previous research has demonstrated that the experience of residing with host families may yield positive or negative outcomes contingent upon the established relationships, influencing the shared time and dynamics of interactions between family members and students (Lafford and Collentine, 2006). Extensive evidence has indicated that cooperative roles adopted by host families facilitate high-quality interactions, allowing students to practice language skills, receive corrective feedback, and acquire new insights, thereby positively impacting linguistic and cultural knowledge (Knight and Schmidt-Rinehart, 2002; Schmidt-Rinehart and Knight, 2004; McMeekin, 2006). Conversely, Magnan and Lafford (2012) highlight factors such as limited patience to communicate with students having lower language proficiency, time constraints due to schedule disparities, interpersonal incompatibility, or stressful coexistence, negatively affecting the learning process. In these studies, individual factors such as language proficiency and personality, along with contextual elements encompassing host family dynamics, are often considered as working independently on students' development of intercultural competence. However, the PPCT design seeks to surpass this simplistic additive effect by investigating the synergistic impact of diverse individual and contextual factors. Within this design, subjects can be concurrently stratified across both personal and contextual dimensions. For instance, different levels of student language proficiency could be matched with variations in host family dynamics, including experiences in intercultural encounters, cultural exchange opportunities, and family routines. Proximal process, in this context, delineates the interaction between students and host families, gauged not solely by the depth and degree of mutual engagement in conversations but also by their frequency and consistency, thereby embedding a Time factor. Furthermore, employing a pre-test and post-test design would allow for the observation of development over time.

The bioecological model can provide hypothetical outcomes of implementing such a design. According to the bioecological model, the potency of proximal processes is intricately tied to the characteristics of the developing individual, the environment, and the specific developmental outcome under scrutiny. Building on Small and Luster (1990) findings, Bronfenbrenner and Morris (2006) posited a hypothesis: for developmental outcomes of *competence*^4^, proximal processes exert the strongest influence within the most advantageous ecological niches. Therefore, a speculative outcome from the proposed research design suggests that student-host interaction might yield the greatest positive impact within favorable host family dynamics, particularly among students displaying the highest language proficiency. This proposition underscores the notion that same levels of host family dynamics may not yield identical effects across all students. Neglecting this distinction disregards the potential for students with superior language skills to benefit more from high-quality student-host interaction, perhaps due to their ability to utilize richer language resources during such interactions. Practically, these hypothetical findings imply diverse intervention strategies tailored to students with varying language proficiency levels to optimally allocate resources within study abroad programs. For instance, interventions targeting students with higher language proficiency might emphasize engaging in proximal processes that demand advanced language skills. Conversely, students with lower language proficiency could benefit from focused support to match them with host families known for patience and assistance in language development. This approach would ensure maximum benefit from proximal processes aligned with their proficiency levels.

These hypothetical implications derived from the PPCT model in the two examples above await empirical validation through future research endeavors. Nonetheless, this illustration posits that the PPCT framework presents distinct advantages in both international and intercultural research and practical application domains. Firstly, it helps enhance predictive precision. By comprehensively analyzing the intricate interactions between multiple factors, the PPCT model offers greater predictive accuracy regarding the effectiveness of interventions or strategies across diverse scenarios or individuals. Secondly, it offers a holistic understanding of development. Embracing personal attributes, contextual elements, and developmental processes across time, the implications derived from the PPCT model encourages holistic approaches to educational interventions. Thirdly, it can inform tailored interventions. The PPCT model facilitates the identification of synergistic relationships among variables, enabling researchers to craft interventions precisely tailored to specific contexts or individuals. Finally, it can also help optimize resource allocation for maximum positive outcomes.

Another methodological implication is that to enhance the depth and richness of insights, future studies can diversify their data collection and analysis methods by incorporating both quantitative and qualitative approaches. Table 2 shows that the research reviewed in this study relies largely on qualitative approaches. However, Navarro et al. (2022) provide some useful illustrative examples of how both quantitative and qualitative researchers can utilize bioecological theory and PPCT. Jaeger (2017) recommends that a hierarchical linear modeling analysis might best approximate Bronfenbrenner's preference for research since it considers a wide range of complex variables for development. In addition to traditional qualitative methods, researchers can also consider employing the qualitative comparative analysis (QCA) method, which offers a systematic approach to analyzing complex causal relationships by identifying configurations of conditions that lead to specific outcomes. It is particularly appropriate for capturing the complexity of Bronfenbrenner's theory and allows researchers to identify patterns and combinations of conditions that are necessary or sufficient for certain educational outcomes. For example, they can examine how different combinations of individual characteristics, environmental factors, and developmental processes interact to influence educational experiences in diverse cultural settings.

Furthermore, to foster innovation in methodological design, researchers can draw inspiration from other disciplines to expand the methodological toolbox in the field of international and intercultural education. For instance, employing biomarkers as a measure of physiological responses (Yrttiaho et al., 2021) within the bioecological framework offers interesting possibilities for future research (Navarro et al., 2022) and could provide valuable insights into the biological underpinnings of individuals' adaptation and development within diverse cultural contexts. Such innovative approaches can offer unique perspectives and contribute to a more holistic understanding of the complex interplay between individual, culture, and education. These methodological considerations can inform researchers to advance the field, deepen our understanding of educational experiences in diverse cultural settings, and contribute to evidence-based practices that promote positive educational outcomes for individuals in intercultural contexts.

In conclusion, this article has reviewed studies utilizing Bronfenbrenner's ecological theory in the context of international and intercultural education. These studies have demonstrated the value of employing this theoretical framework to understand the complex interactions between individuals and their environments in diverse cultural contexts. While some studies have focused on the early version of the theory, others have recognized the more recent bioecological model. Moving forward, it is crucial for researchers to specify the version of the theory they are adopting and to consider incorporating the mature version of the PPCT model. Additionally, future research should explore innovative methodological approaches to gain a comprehensive understanding of the intricate dynamics at play in international and intercultural education.

## Author contributions

PT and IA designed the study and discussed the findings collaboratively. PT was the main contributor for drafting the manuscript. IA contributed extensively to the revised version. All authors agreed on the final version of the manuscript.

## Footnotes

^1^As will be elucidated in more detail in this paper, Bronfenbrenner's theory evolved from an early version of the ecological systems theory to a bioecological model. The general term “ecological theory” is used to encompass both the early version and its recent reformulation of the bioecological paradigm.

^2^In Bronfenbrenner (1986a, 1989)'s earlier theorization, the chronosystem represents a particular type of research design, which should not be confused with the various environmental systems differentiated in his 1979 monograph. However, in his 1994 work, chronosystems are treated as a fifth systems parameter that “extends the environment into a third dimension” (p. 40). Thus, a full representation of Bronfenbrenner's theorization of ecological systems as contexts of development encompasses the four-layered systems conceptualized in 1979, as well as the chronosystem in his subsequent works.

^3^Bhowmik et al. (2018) did not explain why they chose “socioecological model” over more established terms. This lack of clarification leaves room for various interpretations. It might suggest a focus on social aspects within the ecological framework or a departure from the strict bioecological perspective. However, without the authors' explicit explanation, it is hard to determine their intent or whether it is a misapplication. Therefore, clear terminological justifications are crucial, especially when diverging from recognized theoretical labels. This absence of clarification might lead to misunderstandings or ambiguities in understanding Bronfenbrenner's theory and its adaptations.

^4^Development, as per the bioecological model, serves as a neutral term encompassing both positive changes (*competence*) and negative changes (*dysfunction*) (Bronfenbrenner and Morris, 2006). The hypothesis regarding the developmental outcome of dysfunction differs from that concerning competence. For instance, in Drillien's study, proximal processes had their greatest buffering effect on development of problematic behaviors of infants in the most disadvantaged environment but on the healthiest infants (Bronfenbrenner and Morris, 2006).

## Appendix

### Search strategy for WoS and ERIC databases

#### Search strategy for WoS databases

Boolean: (TS=(Bronfenbrenner OR bioecological OR ecological systems theory OR process–person–context–time OR PPCT)) AND TS=(international OR intercultural OR study abroad OR exchange OR mobility OR overseas).

**Filters:**

- Document Types: Article
- NOT Document Types: Proceeding Paper or Book Chapters
- Languages: English
- Web of Science Index: Social Sciences Citation Index (SSCI); Emerging Sources Citation Index (ESCI); Science Citation Index Expanded (SCI-EXPANDED); Arts and Humanities Citation Index (AandHCI)
- Web of Science Categories: Development Studies; Education Educational Research; Education Scientific Disciplines; Education Special; Psychology Education; International Relations; Language Linguistics; Linguistics; Multidisciplinary Sciences; Psychology; Psychology Applied; Psychology Biological; Psychology Clinical; Psychology Developmental; Psychology Educational; Psychology Experimental; Psychology Multidisciplinary; Psychology Social; Social Issues; Social Sciences Biomedical; Social Sciences Interdisciplinary; Social Work; Sociology.

#### Search strategy for the ERIC database

Boolean: (Bronfenbrenner OR bioecological OR ecological systems theory OR process–person–context–time OR PPCT) AND (international OR intercultural OR study abroad OR exchange OR mobility OR overseas).

**Limiters:**

- Peer Reviewed
- Journal or Document: Journal Article (EJ)
- Publication Type: Journal Articles
- Language: English.
